# Hand-Made Embroidered Electromyography: Towards a Solution for Low-Income Countries

**DOI:** 10.3390/s20123347

**Published:** 2020-06-12

**Authors:** Samuel Pitou, Brendan Michael, Karina Thompson, Matthew Howard

**Affiliations:** 1 Centre for Robotics Research, Department of Engineering, King’s College London, London WC2R 2LS, UK; brendan.michael@kcl.ac.uk (B.M.); matthew.j.howard@kcl.ac.uk (M.H.); 2 Craftspace, Birmingham B12 0DU, UK; karina@karinathompson.co.uk

**Keywords:** surface electromyography, textile electrodes, smart textile

## Abstract

Surface electromyography is used for non-invasive evaluations of the neuromuscular system and conventionally involves electrodes placed on the skin to collect electrical signals associated with muscle activity. Recently, embroidered electrodes have been presented as a low-cost alternative to the current commercial solutions. However, the high cost of equipment used in their fabrication forms a barrier to deployment. To address this, this paper presents the first study into the *hand-sewing of electrodes for surface electromyography* to assess its feasibility as an affordable, alternative means of production. In experiments reported here, batches of hand-sewn electrodes from six novice embroiderers are tested for (i) manufacturing consistency, and (ii) myographic data acquisition against conventional gelled and machine-sewn electrodes. First, the electrical properties of the created electrodes are assessed through simple resistance measurements. Then, linear regression is performed using electromyography data to test if force-variation detection is feasible. The results demonstrate that hand-sewn electrodes provide similar sensitivity to force variation as their machine-sewn counterparts according to the linear regression gradients calculated (8.84 using the hand-sewn electrodes and 9.38 using the machine-sewn electrodes, on the flexor muscles of the forearm). This suggests that hand-made, low-cost textile interfaces could be deployed using local production in developing economies.

## 1. Introduction

Surface electromyography (SEMG) is a technology used in the diagnosis, treatment and management of physical disabilities. SEMG is a non-invasive technique used for sensing the electrical activity of targeted muscles through electrodes placed on the skin. It allows the measurement of motor unit recruitment during activity and has a number of clinical applications such as the characterisation of pathologies of the neuromuscular system [1], the assessment and rehabilitation of motor impairment following stroke [2] or the intraoperative monitoring of neuromuscular function to prevent peripheral nerve injury [3].

With the global population currently aging at an increasing rate, demographic studies in developing world posit that the expected number of stroke victims is going to raise in the future [4,5]. It is reported that 91.4 million people in the developing countries are disabled due to stroke compared to 21.5 million in developed countries [6]. These alarming reports show the increasing need for healthcare infrastructure [7] in these settings.

At present, in the developed world, two types of systems are commonly used for SEMG, namely, that use: (i) *dry electrodes* that consist of rigid metal electrodes combined with sophisticated signal processing techniques to gain a signal; and (ii) *gel electrodes* that consist of disposable, adhesive patches. High-end, commercial dry-electrode systems(e.g., Delsys Trigno (Delsys Inc., Natick, MA, USA) or Biometrics Datalite EMG systems (Biometrics Ltd., Ladysmith, USA)) provide highly reliable measurements. They are also reusable and often come in convenient packages (e.g., combining wireless data transmission with sophisticated data acquisition and signal processing software). However, the cost per sensor for such systems is typically around £500–£1000 making them unaffordable in a developing world context.

Recent studies have shown the possibility to create low-cost and efficient SEMG data acquisition systems (consisting of an amplifier and wireless transmitter) for only £60 [8], which is already more affordable than commercial solutions. However, this price does not include the cost of electrodes, which can add a significant increase in cost. If non-gelled commercial reusable Ag/AgCl are used, the price is approximately £11 per electrode, which is a cost barrier to workers in developing economies (equating to approximately four days of work [9]). On the other hand, disposable, adhesive, pre-gelled electrodes (e.g., Ag/AgCl Covidien Kendall disposable electrodes (Medtronic, Minneapolis, MN, USA)) are very cheap (approximately £0.26 per unit), but the cumulative cost can still be prohibitive: (i) for individual patients with on-going usage needs; and (ii) for healthcare providers supplying the needs of a population. For example, the consumable cost of 12 sessions of SEMG biofeedback rehabilitation for a single patient is approximately 15 times higher with gel electrodes than with embroidered textile electrodes (considering that they are re-used over the sessions). For all of these systems, there are also hidden costs that often hinder use in a low-income country setting. For instance, attachment of multiple electrodes to the body to achieve targeted muscle measurements is laborious, and usually requires the skill of a professional healthcare worker. This can be exacerbated by factors such as perspiration, that can interfere with adhesion and conductivity causing poor electrical contact [10]. Both kinds of system also typically require sophisticated machinery and infrastructure (including a stable supply of electricity) for manufacture and qualified staff to operate manufacturing machinery.

Recent research into smart textiles has resulted in a more affordable alternative to these traditional SEMG systems [11]. Specifically, the use of conductive yarns embroidered onto a fabric substrate has been seen to be effective in creating low-cost, flexible and reusable electrodes, suitable for SEMG. The first systematic analysis of the design of such embroidered electrodes was reported in [12], showing that they can achieve near-identical performance to gel-electrode systems. Three design variables affecting the electrical properties of the embroidered electrodes are identified: the dimension of the electrode, the thread spacing and the number of iteration (i.e., embroidering an additional layer of the design on top of an existing embroidery). A grid pattern for sewing with different length of thread has been investigated in order optimise the manufacturability of the electrodes without compromising their performance. Using this pattern, the resulting configuration of the conductive thread is similar to a woven material embroidered onto a fabric substrate. In [13,14], the electrical characterisation of different anisotropic conductive textile materials (such as knitted or woven material) is investigated in accordance to the Van der Paw method modified by Wasscher. The surface resistance $R_{s}$ of a sample is defined such as $R_{s}=\rho /h$ where *h* is the thickness and ρ is the resistivity of the anisotropic sample. However, it is highlighted that the surface roughness has an impact on the electrical properties of the material. In addition, contact resistances occurs in woven structures, adding more complexity to the electrical characterisation of conductive materials.

The machine-embroidered electrodes suffer prohibitive fabrication costs in terms of equipment and infrastructure: the digital sewing machines used to produce existing electrode designs cost around £4000, and require sophisticated licensed software and computing equipment.

With these challenges in mind, this study proposes a new approach to fabrication of electrodes through simple, *hand-stitching techniques*. It assesses the potential for fabrication of textile SEMG electrodes within a resource-constrained setting, by local people with little or no technical knowledge in the operation or electronics of SEMG systems. Table 1 shows an estimate of the potential cost savings that could be achieved with using such hand-sewn embroidered electrodes (HSE) as compared to machine-sewn electrodes (MSE) and gel electrodes (GE) for electromyography-based biofeedback rehabilitation. It also shows that the material cost of the HSE is lower than the MSE because less thread is used in the fabrication. To these ends, this paper reports a feasibility study into whether: (i) non-technical volunteers can be trained to fabricate electrodes and perform basic tests of their functionality; and (ii) the electrodes are fabricated to a standard that they are effective for use in an SEMG application. For this, the *fabrication consistency and quality* of a total of E=60 HSE produced by a cohort of volunteer subjects ($N_{m}=6$) was evaluated by comparing subjects’ self-evaluation of quality against that of an expert electrical tester. The *functionality and reliability of electrodes* fabricated by the objectively more skilled subjects was then evaluated through an isometric grip force test performed with N=10 participants and compared against state-of-the-art MSE and conventional GE. The results suggest that the skill of hand-making electrodes is *easily learnt by those with no prior experience*, and that electrodes fabricated in this manner show *comparable performance to machine-made electrodes*, suggesting their suitability for developing world use.

## 2. Materials and Methods

This study presents dual experimental investigations into: (i) the training of non-technical volunteers in fabrication of SEMG electrodes through hand-embroidery; and (ii) the functionality of the resultant electrodes for data capture. The following describes the experimental procedure, materials and methods for the two experiments in turn. All subjects gave their informed consent for inclusion before they participated in the study. The study was conducted under the approval of the King’s College London Research Ethics Committee, LRS-16/17-4213.

### 2.1. Electrode Embroidery Training

The first step of the experiment assessed the abilities of non-technical workers to hand-craft textile electrodes to a functional standard. For this, volunteers were invited to participate in a pair of one-day workshops to learn the necessary skills. In the results presented here, the experimental participants ($N_{m}=6$) consisted of women without prior professional sewing or electrical testing experience, recruited through the Birmingham-based Shelanu organisation, a charity working to provide opportunities for economic integration of migrants. Within this group, some participants were not fluent in English or born in an English-speaking country.

During the workshops, the participants were taught how to sew the electrodes and how to perform electrical tests in the presence of two workshop facilitators—an embroidery professional and a researcher with background in smart textiles. The workshops were structured so that, in the first workshop, participants were given the opportunity to gain a basic familiarity with a variety of hand-sewing techniques and the fundamentals of the electrical testing required. This ‘basic training’ workshop controlled for the potentially different levels of prior experience in sewing/electrical work of the participants. Following this, in the second workshop, participants were asked to fabricate and test electrodes in a way that simulates independent production in a developing world context. Only electrodes from the latter workshop were used in the SEMG functionality tests (using Section 2.2).

In both workshops, participants used multimeters for resistance measurements, embroidery hoops and support fabric (Vilene, white color, 100% polyamide) with pre-printed electrode template, haberdasher’s snap fasteners, electrical test teaching materials and report sheet (see Supplementary Document S2), needle with conductive thread and sewing teaching materials (see Supplementary Document S1). The embroidery yarn is a stainless-steel conductive thread (Sparkfun DEV-11791, $91.8\;\Omega m^{-1}$). It is a two ply thread spun from a grade 316 L stainless steel fibre (fibre diameter: 8 um, elongation rate: 1.60%). This was used to sew the electrodes and attach a haberdasher’s snap fastener (KIN, 13 mm, nickel-brass rust-proof fastener) to the *top side* of the electrode (i.e., the non-conductive side of the electrode facing away from the skin) to make the connection to a data acquisition device.

The electrical testing required of the participants is a simple measurement of the resistance of the electrode using a multimeter (Excel xl830l with nickel-plated copper probes) making sure that the electrode is always flat on the table. This measure was chosen since the level and inter-electrode consistency of the resistance is an indicator of the expected quality of the SEMG signal: having lower resistance and matched impedance between pairs of electrodes leads to better sensitivity and filtering of noise [18]. Consistency in manufacture has been shown to be an issue in machine-made embroidered electrodes, hence it can be expected that similar issues might arise in hand-embroidery, and is therefore something that a potential developing-world producer should be able to test in their own work for quality control purposes. As a simple quality check, the measurement chosen here is the resistance between the electrode-skin interface (i.e., in the middle of the sewn pattern face) and the metallic snap fastener (above the stud).

#### 2.1.1. Workshop 1: Embroidery with Assistance

The first workshop was designed to give all participants a basic training in sewing and electrical testing, to ensure they all start from the same level of knowledge. For this, several different sewing techniques and electrical tests were introduced to the participants with the help of two workshop facilitators and a set of printed teaching materials (see Supplementary Documents S1 and S2). A demonstration of the sewing techniques and electrical tests was given by the facilitators at the beginning of the workshop and they remained present throughout the session for any participant who requests further help.

**Figure 1:**
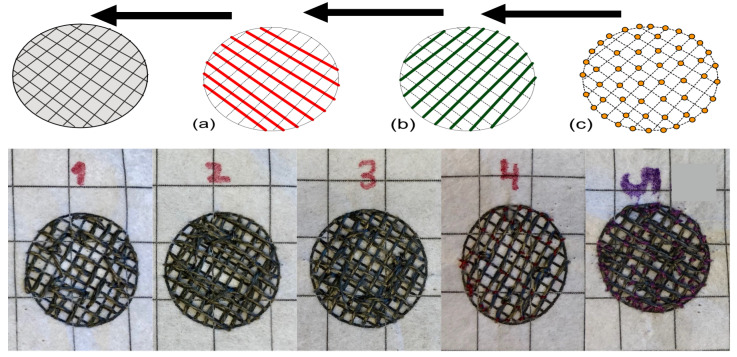
Sewing patterns for electrode fabrication (top row) and typical examples of hand-made electrodes (bottom row). Participants were asked to follow sewing patterns (top row) to learn five techniques for electrode fabrication. To achieve the different techniques, the conductive thread has to be sewn first in (**a**) the red line direction, and then in (**b**) the green lines direction. The yellow dots shown in (**c**) indicate the location of the conductive thread fixations for the couching techniques. Shown are (1) running stitch, (2) float stitch, (3) darning, (4) couching (single line), (5) couching (double line). Detailed sewing instructions are available in Supplementary Document S1.

The participants were taught to hand-sew electrodes according to the same specifications of the best performing machine-embroidered electrodes presented in [18]; the design variables were set so that the electrode have a circular shape with a 2 cm diameter and filled with a grid pattern with a 2 mm spacing. Five sewing techniques were taught to participants to produce these: (i) running stitch; (ii) float stitch; (iii) darning; (iv) single-line couching; and (v) double-line couching. Figure 1 shows the sewing patterns used for each technique. These techniques have been proposed by the embroidery professional as five different ways to hand-sew electrodes for investigating how the design variables affect the electrodes. For example, the running stitch was investigated because it is the technique that uses a shorter length of thread following a grid pattern while the float stitch and darning use more thread length. In addition, both couching techniques were investigated to see if the grid pattern would allow for better electrical properties while using a longer length of thread (in the double-line variation). Other variations between the techniques were different fixations of the conductive thread on the surface of the electrode. Note that, to facilitate learning, each subject was also provided with a piece of support fabric with the desired electrode shape and fill pattern printed on it as a template.

After the participants produced five electrodes (one per sewing technique), the facilitators showed them how to operate the multimeter and perform the desired resistance measurement as an electrical quality check. At the end of the workshop, feedback was sought from participants on their preferred sewing technique for independent fabrication of a batch of electrodes in the second workshop for later use in functional tests. In the experiments reported here, the group chose the float stitch technique, being perceived as the easiest technique to perform accurately.

#### 2.1.2. Workshop 2: Embroidery without Assistance

During the second workshop, the same participants were asked to hand-craft electrodes using their chosen sewing technique (float stitch) with the teaching materials only. As a reminder to participants, the float stitch technique and electrical tests were presented by the facilitators once at the beginning of the session. The participants were then asked to work independently to create a batch of electrodes for functional testing. Each participant was asked to produce 10 electrodes.

To assess participants’ ability to independently test the electrical properties of their electrodes, each participant was asked to report 10 resistance measurements per electrode using the testing technique learned in the first workshop. After the workshop, these measurements were repeated by an expert electrical tester (workshop facilitator) for comparison against the participants’ reported measurements. Finally, the electrodes produced in this workshop were collected to evaluate their use in SEMG measurement (using Section 2.2).

#### 2.1.3. Data Analysis

To assess the participants’ skill in fabrication and electrical testing, the mean and standard deviation of the resistance measurements reported by each participant for the electrodes they created (i.e., *reported measurements*) was computed. For comparison, the same measures were computed on the measurements found by the expert tester (i.e., *verified data*) on the same set of electrodes. These values were compared for each participant. An ANOVA test was used to determine whether the mean resistance value reported (by a specific participant) varies significantly or not from the mean resistance values found by the expert tester on the same electrodes.

To compare the electrical properties of the HSE and the MSE, a test of equivalence was performed on the electrical test data from the expert [19]. The null hypothesis was that the difference between the mean resistance of the HSE (created by a specific participant) and that of a set of 10 MSE is at least 10% of the mean MSE resistance. The alternative hypothesis was that the difference lies within that equivalence interval (i.e., between [−δ,δ]=±10% of the mean MSE resistance). Equivalence was concluded if the confidence interval for α=0.05 fell within the equivalence interval.

### 2.2. Functional Evaluation of Electrodes

The second step of the experiment assessed the functionality of the hand-made electrodes for SEMG data acquisition. As a simple evaluation, an isometric gripping test was chosen, in which subjects applied varying levels of grip-force while the muscle activity in the forearm is measured. From this, linear regression was performed on SEMG data at different force levels for each participants and the gradients, coefficient of determination and sum of squared residuals were analysed. In addition, the coefficient of variation of SEMG grip data within-session was compared for each participants as a simple reliability test of SEMG metrics. Using these tests, the feasibility of using HSE as an SEMG data acquisition interface could be assessed and compared against: (i) a conventional gel-based approach; and (ii) a state-of-the-art machine-embroidered electrode approach.

#### 2.2.1. Materials

**Figure 2:**
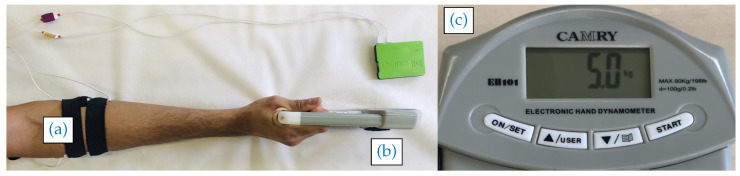
Experimental Setup: (**a**) the textile electrodes location; (**b**) the dynamometer (fixed to the table with tape) and SEMG data acquisition device (in green); and (**c**) the dynamometer value feedback.

Following similar studies in the literature, two channels of SEMG data acquisition were used in the grip-force experiment (see Figure 2). One pair of electrodes was used to measure the flexor muscle group of the forearm (flexor digitorum superficialis), and a second pair measured the extensor muscle group of the forearm (flexor digitorum) [20]. As a result, each trial required the use of four electrodes from those produced in the workshops (two electrodes per SEMG channel). In the context of commercial production in the developing world, the most skilled electrode embroiderers were likely to be the most prolific producers; hence, in the experiments reported here, electrodes made by the participant with the greatest skill in fabrication (as measured by the resistance tests, using Section 3) were used to test functionality. Each trial was therefore conducted using a randomly-selected subset of the electrodes produced by this participant for SEMG capture.

For data acquisition, the *BiTalino (r)evolution Plugged Kit BT* (PLUX, Arruda dos Vinhos, Portugal) was used to sample data from the two SEMG channels synchronously at a rate of 1 kHz via Bluetooth. During data acquisition, signals were monitored by the experimenter to verify good contact with the skin (poor contact is indicated by high-amplitude noise) using the OpenSignal software package (v.2017, Lisbon, Portugal) to plot the time-dependent signal in real-time. A synthetic kinesiology tape and elastic straps (25 mm elastic strap with cam buckle, length: 50 cm) were used to affix the electrodes to the subject’s right arm at the desired skin contact locations. (Note that the tape was used here for the purposes of experimental convenience.)

**Figure 3:**
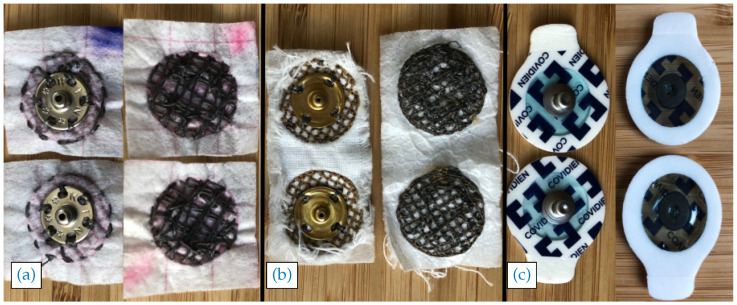
Electrodes used for the experiments. Top and back of: (**a**) the two HSE; (**b**) the two MSE; and (**c**) a pair of GE used for SEMG functionality evaluation. For both types of textile electrode, the back side (consisting of the conductive stainless-steel thread) goes against the skin during recording and the top side (with the snap-fastener) faces outward.

For comparison, the measurements were repeated using MSE and conventional disposable Ag/AgCl GE (Covidien Kendall Arbo H124SG). The MSE were those developed at the Centre for Robotics Research, King’s College London. The latter were created using a Pfaff Creative 3.0 (Pfaff, Kaiserslautern, Germany) programmable sewing machine, employing the design detailed in [18], i.e., the same design used as a template for the HSE (using Section 2.1). This design was chosen as it has been shown to have an optimal trade-off between the electrical properties of the electrodes and their manufacturability. The CAD design for the electrodes was converted to an embroidery file in the 6D Embroidery Software (provided by the sewing machine manufacturer) and was sewn out in fabric using the same stainless-steel conductive thread as that used in the HSE. For attachment to the data acquisition device, the same haberdasher’s snap fastener was sewn to the top side of the electrode, similar to the connections made in the HSE. Examples of the electrodes are shown in Figure 3.

#### 2.2.2. Protocol

The experiment was designed following the theory in [21], where for force isometric exercises, it is expected that the number of motor units recruited change when the grip force and grip duration change. This results in variations in the SEMG signal, and, in particular, increases in grip force are associated with increased SEMG amplitude.

During the experiment, the participant was seated with their forearm on a table and was asked to grasp a dynamometer (Camry 90 kg Digital Hand dynamometer) with their dominant hand without moving the arm for 10 s at five different force levels, with 10 s rest in between. The force values were 5, 7.5, 10 and 12.5 kgf (kgf stands for kilogram-force, 1 kgf = 9.8 N). This exercise was repeated five times for each participant. A rest of 5 min between the exercises was required to avoid fatigue. The whole experiment was performed for each type of electrode to be studied: HSE, MSE and GE. The following reports results for N = 10 subjects with different ages (26.5±2.5 year old), sex (3 women, 7 men), ethnicity, dominant hand (2 left-handed, 8 right-handed) and muscle size.

#### 2.2.3. Signal Post-Processing

During each experiment, raw data from two SEMG channels were recorded. Pre-processing was applied to the data to perform statistical analysis of the signal. The data of each trial were first segmented into equally-sized sections that correspond to a specific force application. To compensate for the short delay between the onset of gripping and the point at which the desired force level was reached, the first 3 s of each sample was discarded (ı.e., the segment size was S=7000 samples) [20]. After that, the following steps were followed to filter and centre the data for each segment.

Denote $e_{t}^{i}\in R^{2}$ as a column vector whose elements consist of the raw SEMG values for the two channels, at the *i*th force level, at time step *t*. The data were first centred around zero by subtracting the mean value of each segment

(1) $$c_{t}^{i}=e_{t}^{i}-{\bar{e}}^{i}$$

where

(2) $${\bar{e}}^{i}=\frac{1}{S}\sum_{t=1}^{S}e_{t}^{i}$$

and S is the length of the segment. A high-pass fourth-order Butterworth filter at 20 Hz as well as a notch filter at 50 Hz was applied to the data in $c_{t}^{i}$ to obtain filtered data [22]. Each element of the latter was then rectified by taking its absolute value, and smoothed by computing a moving average with window size 200 ms. Finally, the average value of each segment of the smoothed data $y_{t}^{i}$ was calculated to associate a single processed SEMG value to a force level

(3) $${\bar{y}}^{i}=\frac{1}{S}\sum_{t=1}^{S}y_{t}^{i}.$$

#### 2.2.4. Data Analysis

First, the reliability of the SEMG data within-session was assessed by computing the coefficient of variation of each segment $c_{t}^{i}$ for each participant to have information on variation in SEMG metrics per force level. Then, linear regression was used to fit a trend line between the SEMG data ${\bar{y}}^{i}$ and force levels, for each channel and participant. In the grip-force task, a linear relationship between the muscle activity and force is expected [23]. The sum of squared residuals *r* and the coefficient of determination $R^{2}$ were computed to assess the fit.

## 3. Results

### 3.1. Electrode Fabrication Training

All participants in the experiment showed the ability to create HSE using the techniques taught in Workshop 1 with varying degrees of skill. Typical examples of the participants’ handiwork are shown in Figure 1. In Workshop 2, six participants were able to create 10 electrodes each, resulting in a total of E=60 electrodes for the electrical and functional tests reported below.

### 3.2. Self-Reported Electrical Tests

**Figure 4:**
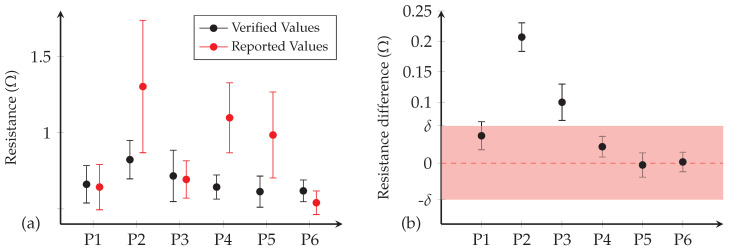
(**a**) Participant-reported and expert-verified resistance of the hand-sewn electrodes (HSE) from each participant; and (**b**) equivalence test comparing the set of electrodes of each participant with the set of MSE. Note: (1) The mean and standard deviation of resistance values over 10 repeated measurements on each of the 10 electrodes created during the workshop. (2) The difference between the mean resistance value of the HSE and that of the MSE (mid-section of the bar) for each participant. The extremities of the bars represent the lower and higher value of the confidence interval. The pink shaded area indicates the extent of the equivalence interval.

The participant-reported and expert-verified resistance of the hand-sewn electrodes created during Workshop 2 are shown in Figure 4a.

As can be seen, the self-reported measurements for three of the participants (i.e., P2, P4 and P5) are considerably higher than the expert-reported ones, and exhibit less consistency (greater ranges of values reported). The results of the ANOVA test indicate that the mean resistance value reported by P2, P4, P5 and P6 varies significantly from the mean resistance values verified by the expert with *p*-value equal to: $p2=4.31\times 10^{-21}$, $p4=1.32\times 10^{-45}$, $p5=1.93\times 10^{-26}$ and $p6=4.41\times 10^{-12}$ for P2, P4, P5 and P6, respectively. However, the participants performed the measurements by following instructions without prior knowledge that contact tips, the measuring force, the flexibility and the surface of measured sample can affect the measured value, which can explain the high standard deviation of the reported resistance measurement. This suggests that these participants did not gain sufficient skills in electrical testing during the two workshops to produce reliable measurements, and that more or improved training in this aspect of the production of HSE may be beneficial. (This also motivates the use of expert-verified data in the further analysis reported below.)

### 3.3. Hand- and Machine-Sewn Electrode Electrical Tests

Figure 4b shows the results of the equivalence test performed on the electrical properties of the HSE and MSE. As can be seen, four of the six participants have created HSE with significantly different electrical properties to those of the MSE (confidence intervals do not cross the zero line and exceed the equivalence interval bounds) with *p*-values: $p1=1.50\times 10^{-3}$, $p2=5.19\times 10^{-33}$, $p3=1.05\times 10^{-7}$ and $p4=9.70\times 10^{-3}$ for P1, P2, P3 and P4, respectively. However, three (P4, P5 and P6) created electrodes with electrical properties equivalent to the MSE according to the 10% difference tolerance bound, rejecting the null hypothesis. Two participants (P5 and P6) created electrodes with no significant difference in electrical properties to their machine-made counterparts with *p*-values: $p5=0.80\times 10^{-3}$ and $p6=0.83\times 10^{-3}$ for P5 and P6, respectively.

Table 2 reports the mean and standard deviation of the verified resistance values for the HSE and MSE. As can be seen, the mean resistance is higher for the HSE produced by participants P1, P2 and P3, and the consistency is lower (higher standard deviation) compared to that of the MSE, suggesting that they would likely to lead to lower quality SEMG capture if used for data acquisition (using Section 2.1). In contrast, the electrodes created by participant P6 exhibited the greatest consistency (lowest standard deviation), even achieving a slightly lower mean resistance than the baseline MSE (albeit with lower consistency). Due to their high standard of production, the electrodes made by this participant are selected for the SEMG functional tests reported below.

### 3.4. Electrode Functional Tests

**Figure 5:**
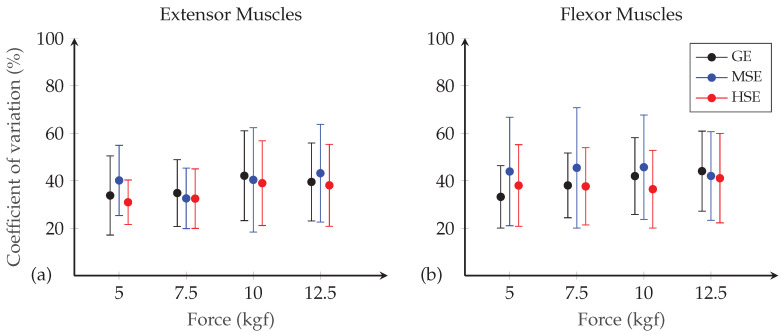
Mean and standard deviation of coefficient of variation (in %) of SEMG data of all participants collected on the (**a**) extensor and (**b**) flexor muscles of the forearm over five trials at different force level for each electrode type.

The result of the reliability test is reported in Figure 5. It can be seen that the coefficient of variation of the SEMG metrics measured with HSE for the extensor muscle group and the flexor muscle group is overall 6% and 2% lower than with GE. In addition, the coefficient of variation of SEMG metrics collected with MSE is approximately 11% higher than with HSE for both muscle groups. These results indicate that the dispersion around the mean of SEMG data is lower for the HSE.

The outcome of the functional tests of the electrodes for SEMG signal capture during isometric gripping over all participants is reported in Table 3. It can be seen that similar values are obtained for the gradient coefficients of the HSE and MSE for the flexor muscle, indicating that variations in force are reflected in the SEMG signal with similar sensitivity. For the extensor muscles, it can be seen that the gradient for the HSE is 25% lower than the MSE, suggesting less (but nevertheless sufficient) sensitivity for detecting force variations. The difference between the two may be explained by the fact that the point on the forearm where the electrodes are placed to monitor extensor muscle activity is usually hairier than that of the flexor group, thereby decreasing the signal to noise ratio.

Looking at the $R^{2}$ values, it can be seen that these are similar between the HSE and MSE for both muscle groups. This suggests that the trend lines have a similar tightness of fit and therefore that the response is reasonably consistent and close to linear, as expected. It should be noted that both the gradient, and the $R^{2}$ are lower for the textile electrodes than for the GE. This might be due to the fact that the textile electrodes are less conductive than the GE, and are more likely to suffer from noise induced by the motion of the skin relative to the electrodes during muscle contraction.

**Figure 6:**
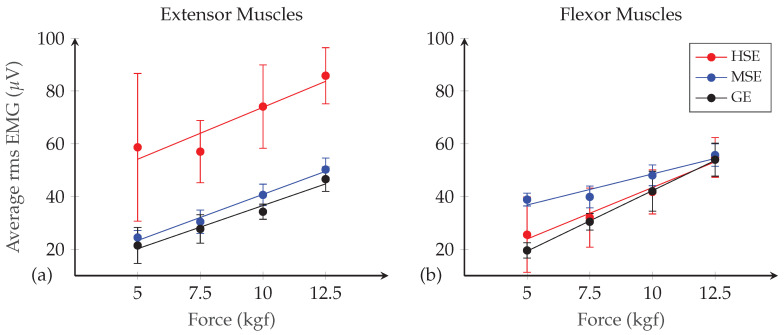
Surface electromyography against grip force measured with hand-sewn electrodes (HSE), machine-sewn electrodes (MSE) and gel electrodes (GE) for a typical participant, tested on the participant’s: (**a**) extensor muscle group; and (**b**) flexor muscle group. Reported are mean values over five trials.

Figure 6 shows the processed SEMG values (${\bar{y}}^{i}$) plotted against force level for a typical participant over five trials. When looking at the results for the extensor group ( Figure 6a), it can be seen that the trend line equation and $R^{2}$ values are similar between the MSE and GE (8.74 gradient and 98% correlation). The results for the HSE show greater sensitivity (a higher gradient of 9.84) but also greater noise (as seen by the higher standard deviation, i.e., bigger error bars). The presence of this noise explains the lower correlation (86%). There is also a noticeable offset in the trend line fit for the HSE. This is not seen in the case of the flexor muscles, so again may be explained by poor electrical contact with the skin on the extensor muscles. The fact that this appears for the HSE and not the MSE may be explained by the fact that the conductive threads in the latter are less tightly couched onto the stabiliser fabric, allowing greater movement with respect to the skin.

In Figure 6b, it can be seen that, for the flexor group, the HSE performance is similar to that of the GE. The gradient of HSE is slightly lower than that of the GE with a value of 9.75 and it can be seen again that HSE exhibit higher noise. However, they have similar correlation values with 97% and 99%. The MSE perform less well according to the trend line equation and $R^{2}$ coefficient reported. Overall, the results of the functional tests indicate that the HSE are viable for use in an SEMG interface to distinguishing variations in grip force.

## 4. Discussion

In this study, an analysis of the fabrication, and electrical and functional properties of hand-sewn embroidered electrodes for SEMG is presented, with a view to their potential use as an affordable, enabling technology in developing-world healthcare systems. Prior work has shown that embroidered electrodes are a promising alternative for affordable SEMG applications, as compared to more traditional gel-based and rigid electrode systems. Their low cost in terms of raw materials and reusability makes them particularly appealing, provided that barriers to their production (cost of equipment and manufacturing infrastructure) can be overcome. To this end: (i) the feasibility of hand-crafting as a means to production (including basic electrical testing) was tested; and (ii) the functional performance of hand-sewn electrodes for muscle activity measurement was assessed.

The results show that, in regard to (i), embroidered electrodes can be created by hand to a similar quality (i.e., with similar electrical properties) as the machine made electrodes. This statement must, however, be qualified in light of the results reported here. First, it appears that hand-crafting of electrodes requires an aptitude to sewing only seen in a subset of the population: in the results reported here, only a half of the participants of the workshops were able to sew electrodes with satisfactory electrical characteristics. This, however, only reflects the fact that people have diverse skills, and it is to be expected that, in commercial production, those exhibiting the greater skill would likely become the more prolific producers. In addition, it is important to notice that the duration of the activity could have impacted the quality of the final products as participant’s engagement could have changed during the workshop. The reported electrical testing suggest that the participants were not able to measure accurately the resistance of the electrodes; one way to improve this would be by providing a range of acceptable resistance values to measure. These results motivate to further the study in the electrical characterisation of the HSE in order to have a precise description of the resistance at the surface of the electrodes. Future resistance measurements for the electrical characterisation of the HSE should be performed in a laboratory following the standards in [24] (making sure the environmental conditions are controlled so that the measurements are performed at 23 °C and 50% humidity), using a methods similar to the ones presented in [14,25].

It is important to consider that, while the training materials and workshops were delivered in English, not all the participants were fluent in English or born in an English-speaking country, and hence may have experienced difficulties in understanding the instructions. For example, a participant who does not apply enough tension to the thread while sewing is more likely to create samples with lower performances due to weak fixations of the conductive thread. This suggests that, had the training been delivered in the participants’ native languages, the proportion of them producing satisfactory electrodes may have been higher. This highlights the need for training tailored to the local production context.

A further point to note is that the population tested in this study had no prior professional experience of sewing or electrical work and received relatively little training through the workshop sessions. Despite this, satisfactory electrodes have been created. It is therefore interesting to consider whether further training, and improved teaching materials, might increase the proportion of participants able to create high quality electrodes. Conversely, this makes the fact that, after such little training, half the population managed to sew acceptable electrodes (i.e., with resistance values equivalent to the MSE) even more remarkable. This may be partially attributable to the fact that textile working is near-universal across countries at all stages of development, hence even if participants have no direct prior experience of sewing, they have some basic level of knowledge about it. This also suggests that, were such approaches deployed more widely for fabricating healthcare devices, there would be a ready population of textile workers to draw upon.

The functional tests in SEMG capture shows that it is feasible to record muscle activity and detect force variation with HSE. In addition, the reliability test shows that the data recorded with HSE are in good agreement with the signal quality of traditional GE. Observations during the experiment can explain that the higher coefficient of variation with MSE is due to the fact that some participants twitched during the exercise of force contraction (to reach the target grip force) after performing trials with the HSE and GE. These cases have not been disregarded in order to keep the same sample size for comparison across participants and gender diversity in the group of participants. In force-variation assessment, the performance of the HSE is lower than that of GE overall, and marginally lower than MSE. This can be explained by several factors in the design and production of the different electrodes. The fact the MSE have two iterations of embroidery [18], whereas the HSE only have one means that the MSE are more likely to have higher conductivity. In addition, the MSE have their conductive thread couched on the substrate material, while the floating stitch design of the HSE means that threads make move relative to the substrate, exacerbating the effects of motion of the electrodes with respect to the skin during muscle expansion during a contraction. This could induce a higher cost of circuitry in order to collect a precise signal, however further training of the participants may enable them to gain skill in producing electrodes using the other sewing techniques suggested here (e.g., double-line couching) to reduce the motion of the threads (and potentially surpass the sewing consistency of the machine). In addition, studies have shown that, even though a lower signal quality is collected through embroidered electrodes, they can be used in application such as monitoring muscle fatigue and decoding muscle patterns in hand prosthesis control [11,26]. Taking these factors into account, the results reported here indicate that the HSE produced by novice participants provide reasonably good performance in SEMG capture. This implies that the HSE may be a solution for affordable SEMG in developing countries, without need for extensive training in their manufacture. Future studies can be performed on a wider population and for a long term use in order to have a generalised view on the performance of hand-sewn electrodes and to verify whether the data do not degrade over time.

## Figures and Tables

**Table 1:** Comparison table of electrode production and SEMG rehabilitation consumables cost for the hand-sewn electrodes, the machine-sewn electrodes and the gel electrodes. The pictures in the table show the equipment for the production of each electrode type. The labour cost estimation is taken from [9,15], representing the daily wage of a textile worker for the hand-sewn electrodes and a machine operator for the gel and machine-sewn electrodes. The estimation of consumables cost is calculated for 12 sessions of rehabilitation using the minimal setup (five electrodes required per session) [16].

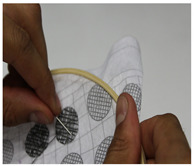	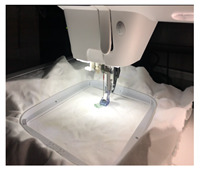	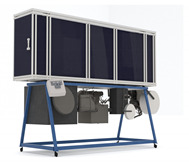
Hand-sewn electrodes production	Machine-sewn electrode production	Gel electrodes production
Raw material cost: £0.14	£0.16	<£0.10 [17]
Equipment cost: £2	£4000	£42,000
Labour cost: £2.51/day	£2.71/day	£2.71/day
Electrode cost: £0.14+£0.07 (labour)	£0.16+£0.08 (labour)	£0.26
Consumable cost: £1.05	£1.2	£15.6
Note: Dry and reusable, easy production in rural area, potential to involve the unskilled population and create employment.	Dry and reusable, good signal quality, electricity reliant production, plant and machine operator required.	Disposable and adhesive electrode, good signal quality, electricity reliant production, plant and machine operator required.

**Table 2:** Comparison of the HSE with the MSE. Mean and standard deviation (SD) of the expert-verified resistance measurements (Ω) over: (i) all the HSE produced by the participants (as indicated); and (ii) the baseline MSE.

Participant	P1	P2	P3	P4	P5	P6	Baseline
Mean	0.66	0.82	0.71	0.64	0.61	0.61	0.64
SD	0.123	0.126	0.168	0.079	0.102	0.071	0.042

**Table 3:** Linear regression values for SEMG data of all participants over five trials.

Type	Extensor Muscle			Flexor Muscle		
	Gradient	$R^{2}$	*r*	Gradient	$R^{2}$	*r*
HSE	9.61	0.76	268	8.84	0.82	98
MSE	12.79	0.75	207	9.38	0.78	138
GE	13.69	0.92	43	13.87	0.97	26

## Supplementary Materials

The following are available online at https://www.mdpi.com/1424-8220/20/12/3347/s1, Document S1: Embroidery teaching materials, Document S2: Electrical test teaching materials.
