# Supplementary material for: Hand-Made Embroidered Electromyography: Towards a Solution for Low-Income Countries
Source: Sensors (Basel). 2020 Jun 12;20(12):3347. doi: 10.3390/s20123347 (PMC7349794; doi:10.3390/s20123347)
Supplement: Supplementary file 1 [file sensors-20-03347-s001.pdf]

Supplementary :

S1_EmbroideryTeachingMaterial

Sewing teaching material

Author: Karina Thompson

Thank you agreeing to help us!

Today we will try out differnet ways to stitch out the electromyography sensor. We have 5 different ways of sewing the sensors. We will be using conductive thread made out of stainless-steel to do this.

You might find some techniques harder or easier. We would like to know which task you found tricky and which task worked best for you.

Materials Needed:

- A piece of stabiliser
- Stainless steel conductive thread, and non-conductive thread
- A needle Darner 1/5
- An embroidery hoop
- Haberdashery snap fasteners

The 5 techniques presented are

1. Running stitch
2. Float stitch
3. Darning
4. Couching (single line)
5. Couching (double line)

Basic top tips

1) First thread your needle with about 30cm of conductive thread.
2) Make a knot at the end of your thread. Pull the needle through the fabric where you need to start so the knot is on the back.
3) When finished, go over the same stitch three times to keep it secure. You could also bring the thread through to the back of the fabric and secure with a knot.

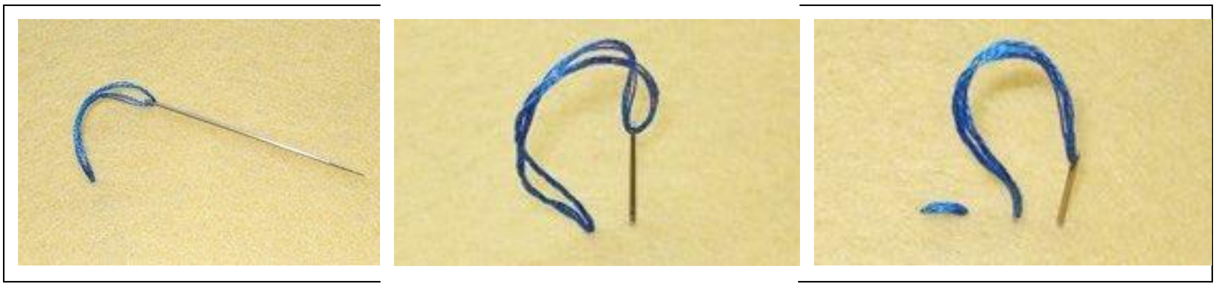

After sewing, each sensor will need a snap fastener stitched to the back of it. If you have enough thread you can carry on and stitch it with that. Alternatively you could stitch it with a new piece of conductive thread. Make sure you put a couple of stitches through each hole of the snap fastener.

<u>1: Running stitch each stitch 2 boxes long</u>

Make each stitch 2 boxes long. Use a stitch to go around a corner like number 5. Work your way across the circle.

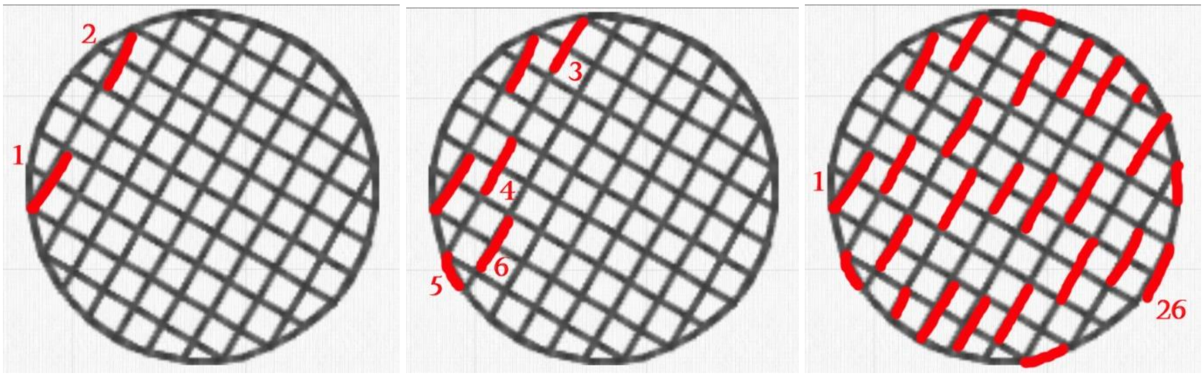

When you have done all the lines one way come back the other way. We have used green to show what your stitches might look like but remember your thread will all be one colour.

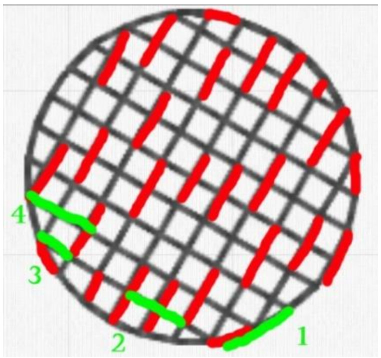

<u>2: Float stitch</u>

This is similar to the running stitch except each stitch is the length of the sensor. Work your way across in one direction and then come back the other way. You might find you need to do some small stitches on the edge to get to the best place to start. The stitches should just sit on top of each other.

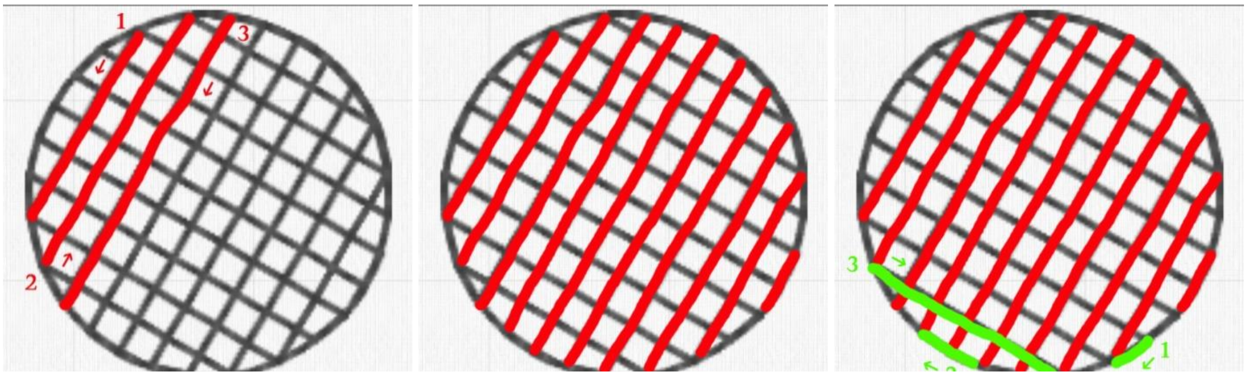

3: Darning

This is nearly exactly the same as the float stitches. Work your way across the sensor with long stitches as before. When you come back the other way weave your needle in an out of the long float stitch (not the fabric, just under and over the thread).

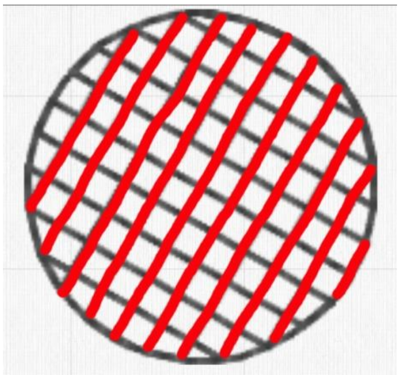

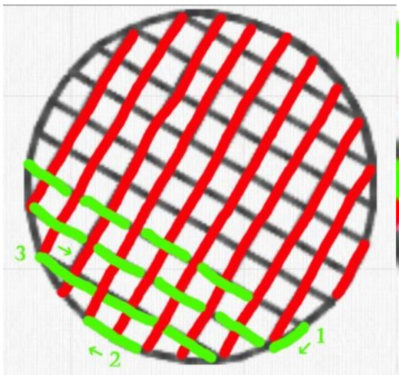

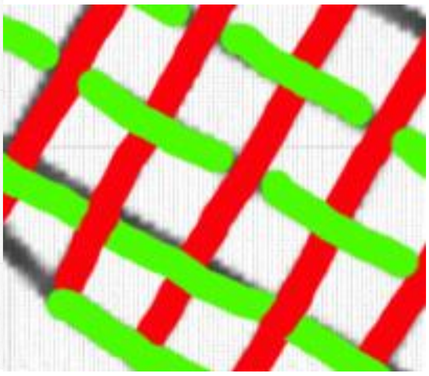

4: Couching (the single line)

Couching is where you stitch a thicker conductive thread down with separate little stitches of non-conductive thread. After you pass the conductive thread through the fabric it doesn't go back through the fabric until you finish. You will need to thread a needle with your conductive thread and a second needle with a non-conductive thread.

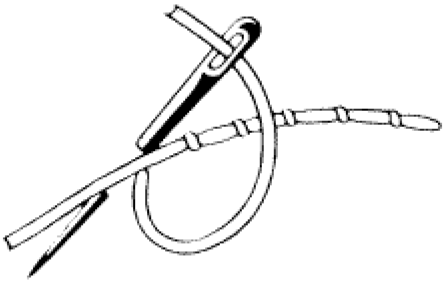

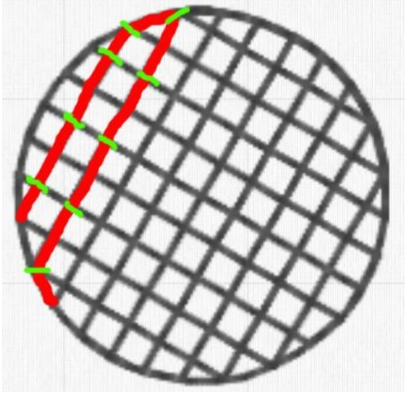	The red line is the conductive thread. The green lines are the smaller stitches that are holding the thicker thread in place. Work your way across the disc and then finish by passing the conductive thread through the fabric and securing it.	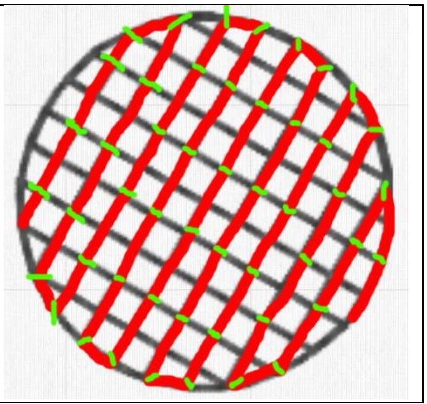

5 : Couching (the double line)

This starts exactly the same as in 4. When you reach the other side work your way back so all printed lines are covered.

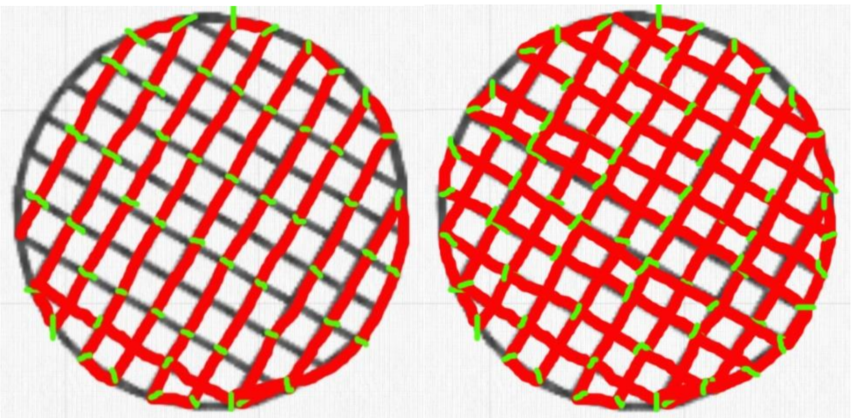

S2_ElectricalTestTeachingMaterial:

Electrical Testing Handout

Your tools :

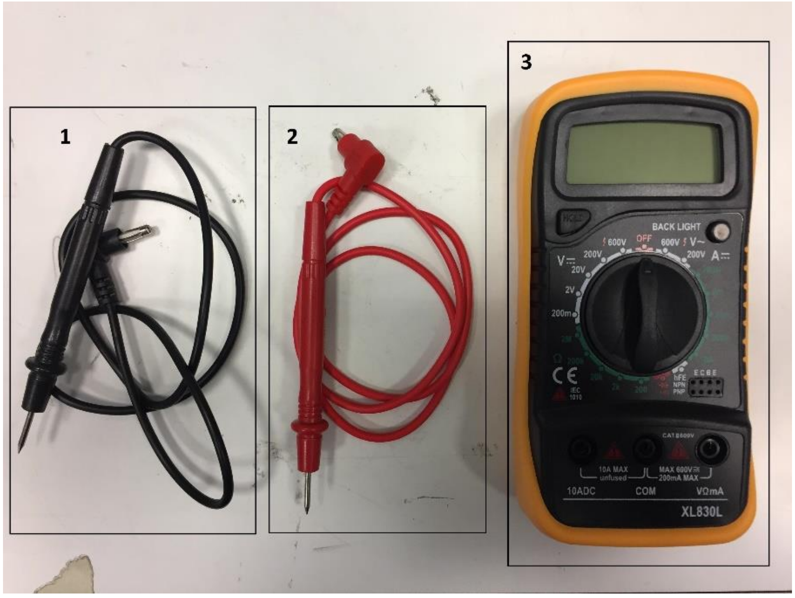

In the box you should find :

1- A Black Cable
2- A Red Cable
3- A Multimeter

Step 1 : Prepare your workspace

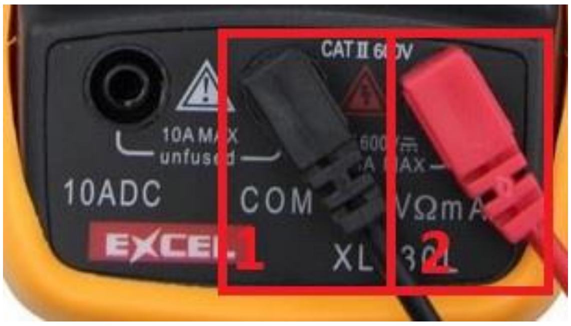

1 – Plug the Black probe in the COM port

2 – Plug the Red probe in the VΩmA port

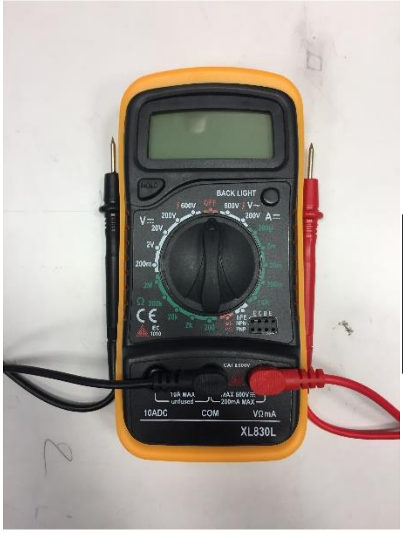

Your workspace should look like this

Note: Make sure that the cables are well plugged – Please double check

Step 2 : Switch on the Multimeter

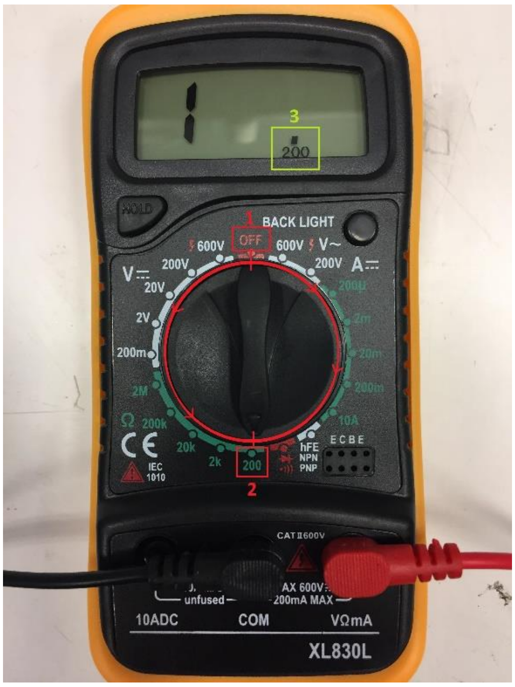

Switch on the Multimeter :

A- Turn the knob from position 1 to 2 to switch on the multimeter and choose the setting.
B- Verify that the setting is correct : the screen should display 200 (3)

Step 3 : Do the measurements

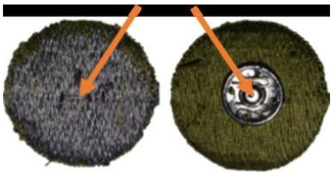

To do the electrical testing you will have to place the probes in the position indicated by orange arrows.

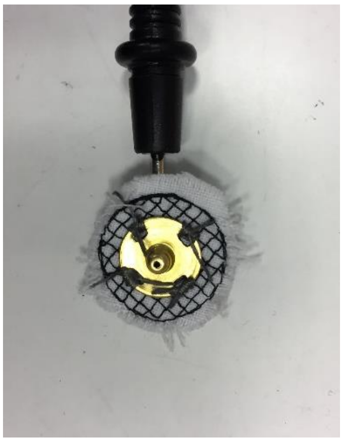

1- Place the <u>Black</u> probe <u>under the electrode</u>

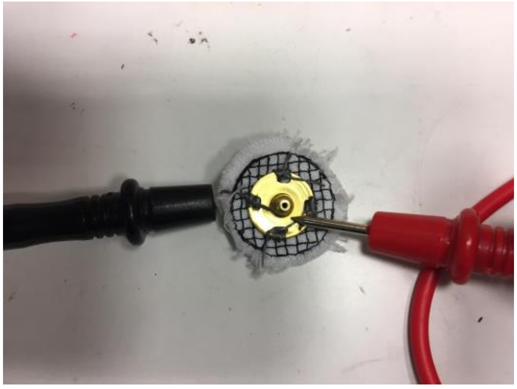

2 – Place the <u>Red</u> probe <u>on the fastener</u>

3- This is a picture of how the electrical testing should be done. The value displayed on screen will have to be reported (see step 4)

Note : Hold each probe with your hands to make sure that the probe is touching the right surface and that the electrodes is stable.

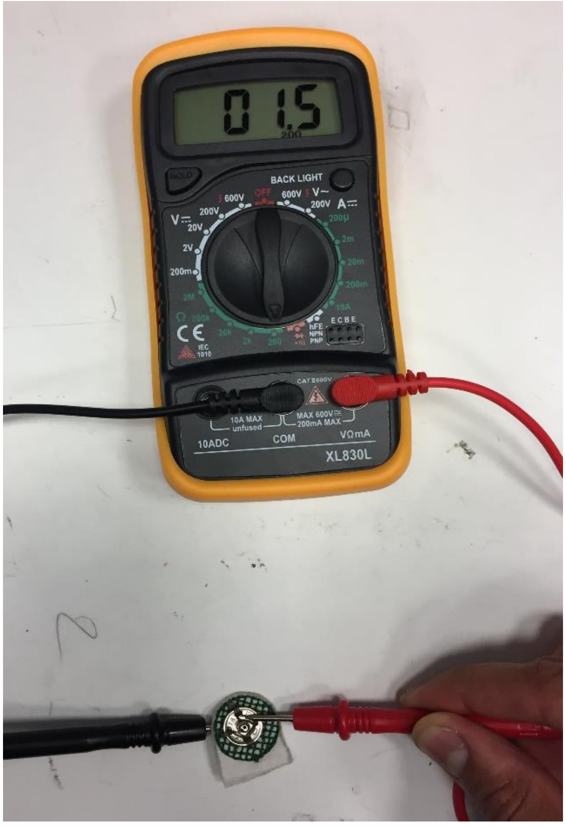

Step 4 : Report your measures

You will have to do **20 measures per electrodes** and report the values in the table below.

Important:
The measured value should be under 20 Ohms.
The values for different electrodes should be similar.
If not, ask a supervisor at the workshop.

Warning:
Hold 1 probe with 1 hand! The probes have to be stable during the measurements, use both hands.
Correct measures are displayed on the right side of the screen.
Incorrect measures are displayed on the left side of the screen. It means that the device has a problem or a wrong setting, please call a supervisor.

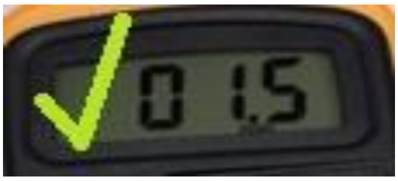

Correct measure

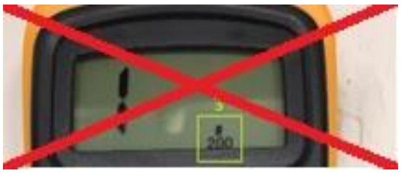

Incorrect measure

M\E	E01	E02	E03	E04	E05	E06	E07	E08	E09	E10	E11	E12
M01												
M02												
M03												
M04												
M05												
M06												
M07												
M08												
M09												
M10												
M11												
M12												
M13												
M14												
M15												
M16												
M17												
M18												
M19												

**M20**												

M: measurement number

E: electrode number
